# Immune checkpoint inhibitor‐induced epidermal necrolysis: A narrative review evaluating demographics, clinical features, and culprit medications

**DOI:** 10.1111/1346-8138.17039

**Published:** 2023-11-30

**Authors:** Takashi K. Satoh, Matthias Munoz Neulinger, Pia‐Charlotte Stadler, Rui Aoki, Lars E. French

**Affiliations:** ^1^ Department of Dermatology and Allergy University Hospital, LMU Munich Munich Germany; ^2^ Dr Phillip Frost Department of Dermatology and Cutaneous Surgery, Miller School of Medicine University of Miami Miami Florida USA

**Keywords:** immune checkpoint inhibitors (ICIs), immune‐related adverse events (irAEs), severe cutaneous adverse reactions (SCARs), Stevens‐Johnson syndrome (SJS), toxic epidermal necrolysis (TEN)

## Abstract

Immune checkpoint inhibitors (ICIs) have transformed cancer treatment but can cause immune‐related adverse events (irAEs). Severe cutaneous irAEs, including epidermal necrolysis, are rare but potentially life‐threatening. There is limited understanding of the clinical features and management of ICI‐induced Stevens‐Johnson syndrome (SJS)/toxic epidermal necrolysis (TEN), so we aimed to analyze 95 cases of ICI‐induced SJS/TEN (35 cases of SJS, 26 cases of TEN, two cases of SJS/TEN overlap, and 32 cases of unspecified) to increase knowledge of this condition among oncologists and dermatologists. We conducted a comprehensive search of PubMed for all relevant case reports published until the end of December 2022, and collected data on patient demographics, cancer type, ICI regimen, time to onset of SJS/TEN, clinical presentation, management strategies, and outcomes. PD‐1 inhibitors were the most common ICIs associated with SJS/TEN (58.9%), followed by the combination of PD‐1 and CTLA‐4 inhibitors (11.6%), and PD‐L1 inhibitors (6.3%). Lung cancer and melanoma were the most frequent malignancies treated (35.8% and 25.4%, respectively). SJS/TEN occurred most frequently within the first 4 weeks (51.7%), and corticosteroid monotherapy was the most commonly chosen systemic treatment (56.4%). The overall mortality rate of ICI‐induced SJS/TEN was 30.8%. Our findings highlight the frequency and severity of ICI‐induced SJS/TEN and the urgent need for predictive molecular biomarkers aimed at preventive measures and early intervention.

## 1 INTRODUCTION

Immune checkpoint inhibitors (ICIs) have revolutionized the treatment of cancer, but their use has been associated with a wide range of immune‐related adverse events (irAEs).^1^, ^2^ While severe cutaneous irAEs are infrequent, they can be fatal or cause long‐term complications.^3^, ^4^ Epidermal necrolysis is a rare but serious and potentially life‐threatening skin reaction triggered by certain medications.^5^, ^6^ This condition is characterized by blistering and skin detachment, and encompasses Stevens‐Johnson syndrome (SJS), toxic epidermal necrolysis (TEN), and SJS/TEN overlap syndrome. These conditions are primarily distinguished by the percentage of body surface area affected, with SJS affecting less than 10%, TEN affecting 30% or more, and SJS/TEN overlap syndrome falling in between. Despite the severity of these conditions, there is a paucity of information regarding the clinical features and management of ICI‐induced SJS/TEN, which includes SJS, TEN, and SJS/TEN overlap syndrome. Therefore, the aim of this study was to increase understanding of this rare but devastating condition among oncologists and dermatologists by analyzing 95 cases of ICI‐induced SJS/TEN. In addition, this study aimed to identify patient background, management strategies, and prognosis associated with ICI‐induced SJS/TEN to aid clinicians in diagnosing and treating this condition.

## 2 METHODS

This retrospective analysis reviewed patients who developed SJS/TEN during immunotherapy treatment. A comprehensive search of PubMed was conducted for all relevant case reports published until the end of December 2022. Inclusion criteria were based on patients who had received ICIs and were diagnosed with SJS/TEN based on clinical presentation. Exclusion criteria included cases that were not related to ICI treatment or had incomplete medical records. Data collected included patient demographics, cancer type, ICI regimen, time to onset of SJS/TEN, clinical presentation, management strategies, and outcomes.

## 3 RESULTS

**Figure 1:**
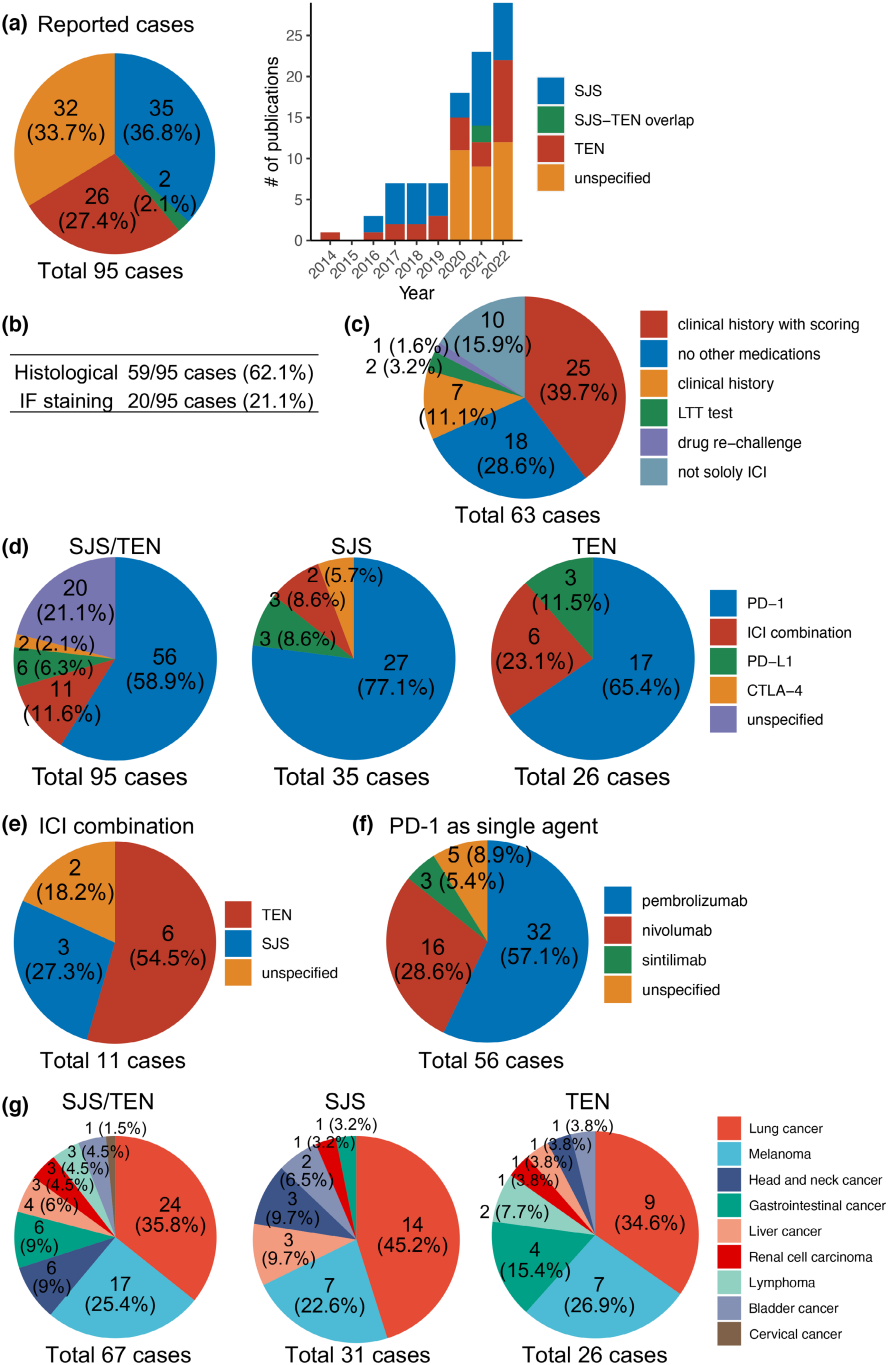
(a) Total number of reported cases of ICI‐associated SJS/TEN, including SJS, SJS/TEN overlap, and TEN, and the number of publications per year. (b) Number of reported cases with histological evaluation and immunofluorescence staining. (c) Diagnostic procedures for ruling out non‐ICI drug‐induced SJS/TEN. (d) Breakdown of ICI therapies that are associated with SJS/TEN subtypes. (e) Breakdown of SJS/TEN subtypes associated with ICI combination therapy. (f) Breakdown of specific PD‐1 inhibitors associated with SJS/TEN as a single agent. (g) Distribution of malignancies for which ICI therapy was initiated in reported cases of SJS/TEN.

In total, 95 cases of ICI‐induced SJS/TEN were identified from 55 peer‐reviewed publications, with 35 cases of SJS, two cases of SJS/TEN overlap, and 26 cases of TEN^7^, ^8^, ^9^, ^10^, ^11^, ^12^, ^13^, ^14^, ^15^, ^16^, ^17^, ^18^, ^19^, ^20^, ^21^, ^22^, ^23^, ^24^, ^25^, ^26^, ^27^, ^28^, ^29^, ^30^, ^31^, ^32^, ^33^, ^34^, ^35^, ^36^, ^37^, ^38^, ^39^, ^40^, ^41^, ^42^, ^43^, ^44^, ^45^, ^46^, ^47^, ^48^, ^49^, ^50^, ^51^, ^52^, ^53^, ^54^, ^55^, ^56^, ^57^, ^58^, ^59^, ^60^, ^61^, ^62^, ^63^ (Figure 1a and Supporting Information Table S1). The precise nomination of the remaining 32 cases as SJS, SJS/TEN overlap or TEN was not specified by authors of the above publications.

Histological findings were described in 59 cases (62.1% of case reports), and the observed features were consistent with those typically seen in SJS/TEN, including interface dermatitis, focal full‐thickness epidermal necrosis, scattered necrotic keratinocytes in the epidermis, and a sparse perivascular lymphocytic infiltrate, as well as a perivascular infiltrate consisting of lymphocytes and eosinophils (Figure 1b). Immunofluorescence staining was performed in 20 cases (21.1%) (Figure 1b).

Sixty‐three out of 95 cases included information regarding the exclusion of drugs other than ICIs as the cause of SJS/TEN. Exclusion was straightforward when only ICIs were administered (18 cases, 28.6%) (Figure 1c). However, in cases where non‐ICI drugs were co‐administered, the most common exclusion method involved a thorough review of medications, medical history, and the evaluation of algorithms such as the Algorithm of Drug Causality for Epidermal Necrolysis (ALDEN) score and the Naranjo Adverse Drug Reaction Probability Scale (Naranjo scale).^64^, ^65^ This method was used in the most frequent cases (25 cases, 39.7%). Subsequently, exclusion without scoring was applied in some cases (seven cases, 11.1%). Additionally, the lymphocyte transformation test (LTT) and drug rechallenge were utilized for exclusion in two and one case, respectively. While four articles have reported SJS/TEN occurring due to the combination with ICI and non‐ICI drug(s) (10 cases, 15.9%), only one case demonstrated potential co‐causality with a non‐ICI drug based on LTT.^29^, ^34^, ^36^, ^63^

Programmed cell death protein 1 (PD‐1) inhibitors were the most frequent type of ICI reported to be associated with SJS/TEN, followed by the combination of PD‐1 and cytotoxic T‐lymphocyte associated protein 4 (CTLA‐4) inhibitors, and PD‐L1 inhibitors (Figure 1d). This observation is likely attributed to the most frequent use of PD‐1 inhibitors among ICIs. Combination therapy with PD‐1 and CTLA‐4 inhibitors was the only combination therapy reported in all cases. More cases of TEN were reported than those of SJS caused by ICI combination therapy, in contrast to the cases resulting from the single use of PD‐1 or CTLA‐4 inhibitors (Figure 1d,e), probably indicating an association between the stronger immunological stimulation by combination therapy and the severity of SJS/TEN. The single agent ICI drugs most commonly associated with SJS/TEN were pembrolizumab and nivolumab (Figure 1f). Two cases that had received irradiation treatment against malignancies experienced the spread of lesions beyond the irradiated areas and subsequently developed SJS following ICI treatment.^23^, ^27^

The most frequent malignancies treated in this cohort of patients developing ICI‐associated SJS/TEN were lung cancer (35.8%) and melanoma (25.4%), followed by head and neck cancer (9%) and gastrointestinal cancer (9%) (Figure 1g). There was no apparent correlation between the occurrence of SJS/TEN and the type of malignancy. The frequency of SJS, SJS/TEN overlap, and TEN was nearly comparable, except for gastrointestinal cancer, which was more frequently associated with TEN (15.4%) than SJS (3.2%).

**Figure 2:**
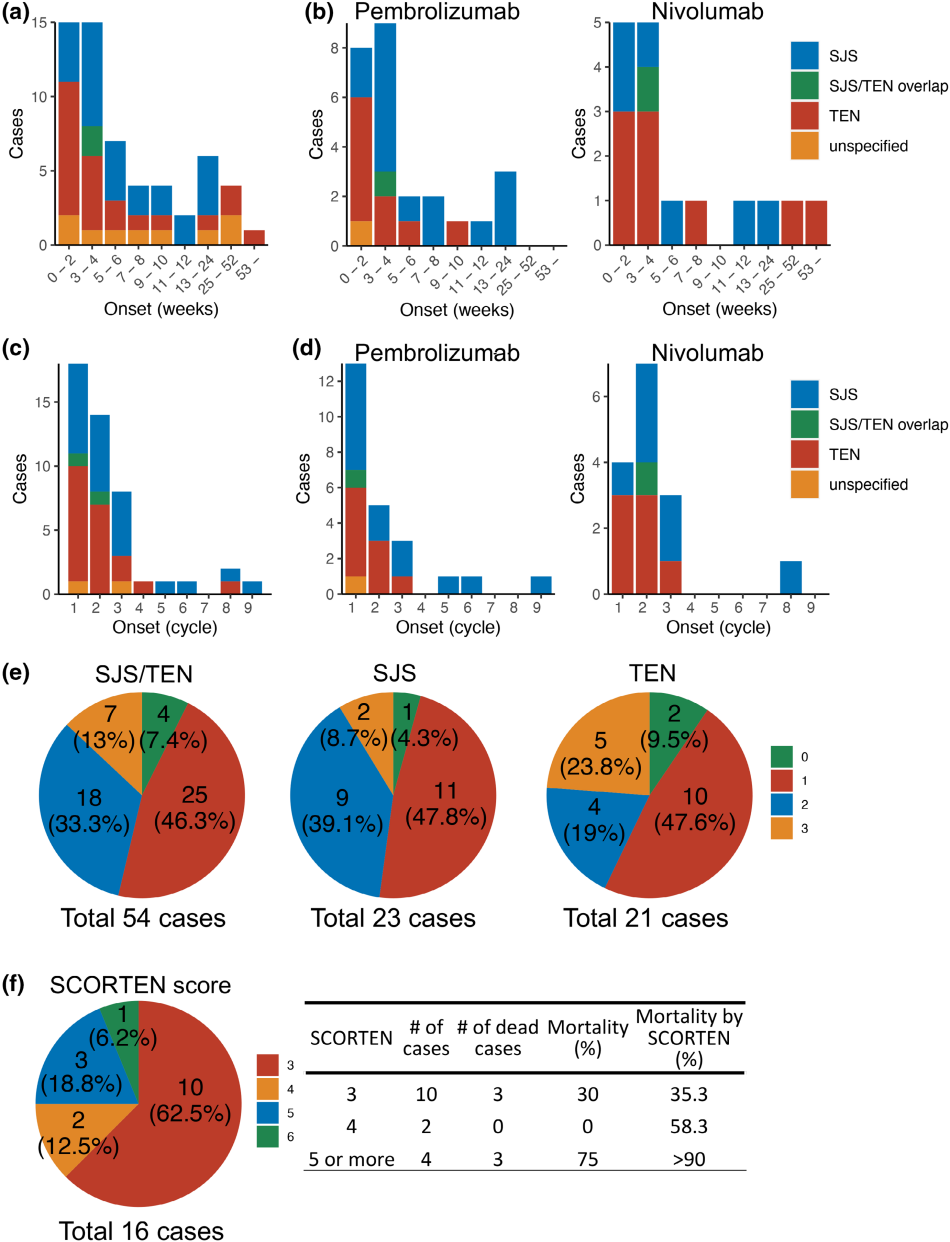
(a, b) Time (weeks) to onset in ICI‐associated SJS/TEN according to subtype. (c, d) Treatment cycle at onset for ICI‐associated according to the subtype. (e) Number of mucosal regions involved in ICI‐associated SJS/TEN, as well as a breakdown according to the subtype. (f) Maximum SCORTEN score observed in patients with ICI‐associated SJS/TEN.

The time to onset of SJS/TEN during ICI therapy was most frequent (30 (= 15 + 15)/58 cases, 51.7%) within the first 4 weeks (2–28 days) after onset of ICI therapy (Figure 2a). Despite this predominance within the first 4 weeks after therapy onset, it is of note that a subgroup representing 19% of SJS/TEN cases (11 (= 6 + 4 + 1)/58 cases) occurred more than 3 months (13 weeks or more) after initiating ICI therapy onset (Figure 2a). The earliest reported onset of ICI‐associated SJS/TEN was on day 2.^29^ The tendency to cause SJS/TEN within 4 weeks was observed similarly with monotherapy of nivolumab and pembrolizumab, which were the most commonly reported drugs (Figure 2b). Thus ICI‐associated SJS/TEN most frequently occurred within the first cycle of ICI therapy, and the frequency decreased thereafter (Figure 2c). There were reported cases observed in later cycles 8 and 9. Of note, the peak onset cycle of SJS/TEN due to pembrolizumab was the first cycle, while that for nivolumab was the second cycle (Figure 2d). Given the variable dosing intervals, being every 2 weeks for nivolumab and every 3 weeks for pembrolizumab, the time of onset of SJS/TEN to PD‐1 inhibitors may be more appropriately reported as the time from the start of administration (within 4 weeks) than the number of infusion cycles.

Mucosal involvement is very common in classical SJS/TEN, occurring in approximately 90% of cases, with typically at least two different sites involved either preceding or following skin eruption.^66^, ^67^ In ICI‐induced SJS/TEN, information about mucosal involvement was available for 54 out of a total of 95 cases (Figure 2e). Among these 54 cases, 50 (= 25 + 18 + 7) cases had involvement of one or more mucosal regions (50/54 cases, 92.6%), while four cases had reported no mucosal involvement (4/54 cases, 7.4%). This indicates that the ratio of ICI‐induced SJS/TEN with at least one mucosal region is similar to that of classical SJS/TEN.^9^, ^34^, ^35^, ^57^, ^66^, ^67^ The most commonly reported number of mucous regions involved in SJS/TEN was one (46.3%), followed by two (33.3%) and three regions (13%) of the evaluated regions (eyes, nasal‐oral, and urogenital areas), as shown in Figure 2e. Involvement of all three mucosal regions was more frequent in case of ICI‐associated TEN than SJS (23.8% vs. 8.7% respectively; Figure 2e).

Of all the cases included within this narrative review, 16 cases (16.8%) included an evaluation of severity using the Severity Score for Toxic Epidermal Necrolysis (SCORTEN) (Supporting Information Table S1). Among these cases, 62.5% (10 cases) had a SCORTEN score of 3, which predicts a mortality rate of 35.3% in the case of conventional drug‐induced SJS/TEN. Among them, the mortality rate was 30% (three cases). Additionally, 25% (four cases) had a score of 5 or higher, which predicts a mortality rate of over 90% in classical SJS/TEN. Among this subgroup, the mortality rate was 75% (three cases) (Figure 2f).

Systemic therapy was chosen for the majority (56/60 cases, 93.3%) of reported patients with ICI‐associated SJS/TEN, while supportive care without systemic therapy was selected for a minority of patients (6.7% of total cases), including two cases of SJS, one case of SJS/TEN overlap, and one case of TEN (Supporting Information Table S1).

**Figure 3:**
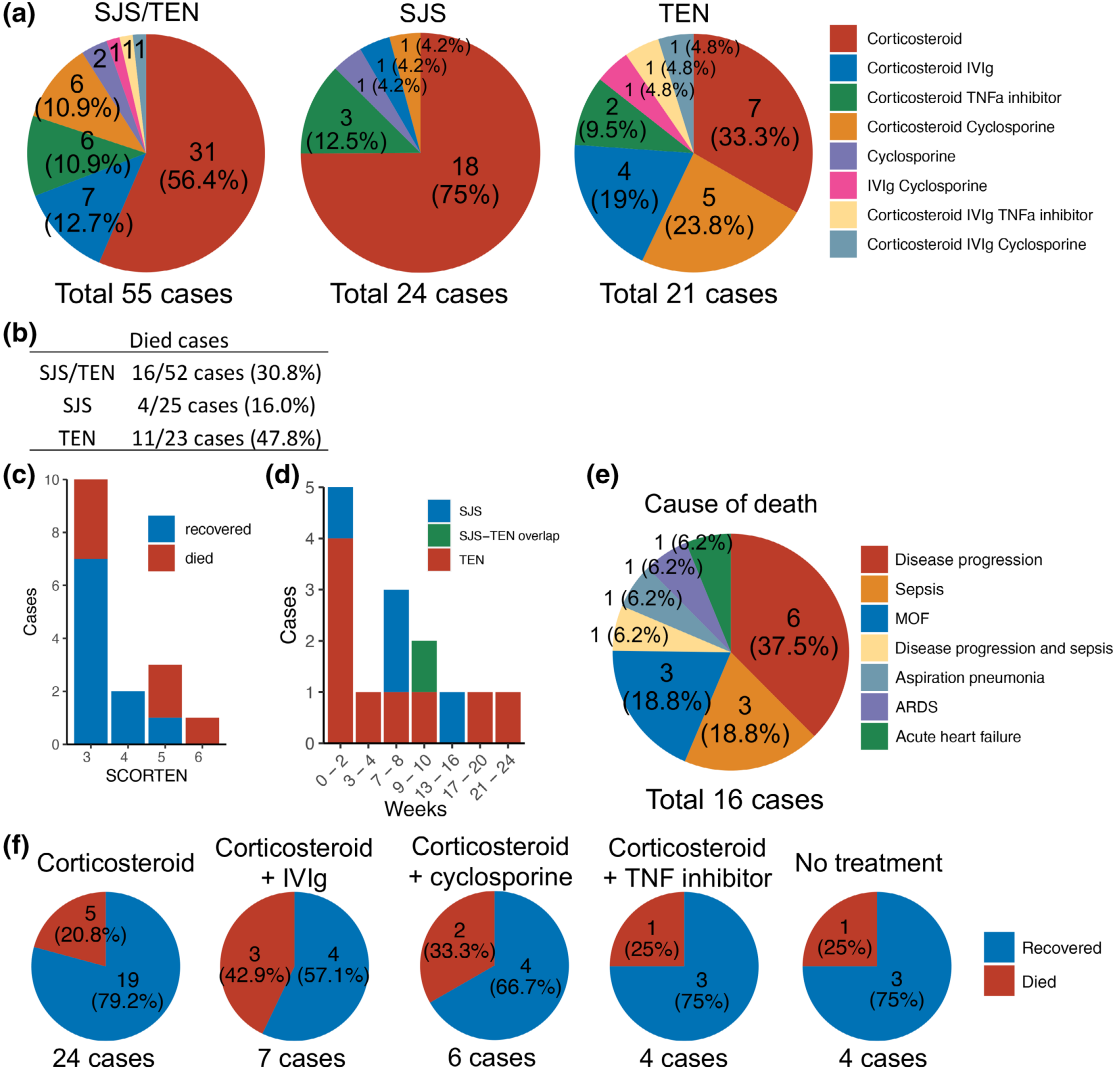
(a) Breakdown of systemic therapies used with respect to ICI‐associate SJS/TEN subtype. (b) Mortality of ICI‐associated SJS/TEN. (c) Mortality and SCORTEN scores. (d) Time (weeks) to death after the onset of ICI‐associated SJS/TEN and breakdown into the subtypes. (e) Cause of death in patients with ICI‐associated SJS/TEN. (f) Mortality with respect to systemic therapy used.

Among the 55 ICI‐associated SJS/TEN cases with available information on treatment, systemic treatment with corticosteroid was the most commonly chosen method (56.4% of cases), followed by a combination of corticosteroid with intravenous immunoglobulin (IVIg) (12.7% of cases), cyclosporine (10.6% of cases), and tumor necrosis factor (TNF) inhibitors (10.6% of cases) (Figure 3a). While the proportion of cases treated with corticosteroid alone was higher in SJS (75%), it was only 33.3% in TEN, where a combination with cyclosporine, IVIg, and TNF inhibitors was more frequently used.

The overall mortality rate of ICI‐associated SJS/TEN in this cohort of patients was 30.8% (Figure 3b). Taken alone, the mortality for ICI‐associated SJS was 16% and TEN 47.8%. Among cases with recorded SCORTEN scores and mortality, a trend towards an association of higher SCORTEN scores and increased observed mortality was observed (Figure 3c).

The duration from onset of SJS/TEN to death was most frequent under 2 weeks (35.7%), with death occurring within 1 week in 21.4% of cases (three out of 14 cases) (Figure 3D).^16^, ^51^, ^62^ The most common causes of death in the context of ongoing SJS/TEN were cancer progression, sepsis, and multiple organ failure (Figure 3e). There was no apparent difference in mortality rates when comparing different treatment methods and types of supportive care, except in the case of corticosteroid monotherapy, where a trend of a lower mortality rate compared to other treatments was observed (Figure 3f). Average mortality rates of corticosteroid monotherapy and all other treatments including no treatment were 20.8% and 33.3%, respectively. This is likely due to the more frequent use of corticosteroids alone in SJS than in TEN, and the predominant use of corticosteroid monotherapy in less severe forms of SJS/TEN (Figure 3a). In this cohort, intensive supportive care without systemic treatment revealed a similar mortality rate (25%) to corticosteroid monotherapy (20.8%) (Figure 3f), but since this result is based on only four cases (two cases of SJS, one case of SJS/TEN overlap, and one case of TEN), further investigation is needed to determine the most effective treatment for SJS/TEN caused by ICIs.

## 4 DISCUSSION

The results of this narrative review evaluating demographics, clinical features, and culprit medications reported for ICI‐associated SJS/TEN highlight important clinically relevant characteristics, the severity of this adverse cutaneous reaction as well as current gaps in knowledge that are relevant for optimal patient‐management. The predominant occurrence of SJS/TEN within the first 4 weeks after the initiation of ICI therapy, along with the lower albeit persistent risk of SJS/TEN at later treatment time points and significant associated mortality, emphasizes the critical need for early and continuous diagnosis and management of this condition.

Furthermore, the higher incidence of severe forms of SJS/TEN, notably TEN characterized by more than 30% body surface epidermal detachment, with ICI combination therapy compared to monotherapy, suggests that the enhanced immune‐stimulation through checkpoint‐inhibition may increase the risk and severity of SJS/TEN. This finding is consistent with previously published data reporting that irAEs in general are more frequent in the patients treated with ICI combination therapy, and that grade 3 or 4 events may lead to discontinuation of ICIs in up to 36% of treated patients.^68^ In addition, it is worth noting that radiation therapy has the potential to enhance immune responses, which may increase the risk of SJS/TEN development. This observation is supported by reported cases of SJS accompanied by radiation recall dermatitis following immunotherapy.^23^, ^27^, ^69^

Diverse cutaneous adverse drug reactions, including maculopapular drug exanthema, drug rash with eosinophilia and systemic symptoms, and “classical” SJS/TEN, which are drug hypersensitivity reaction caused by conventional medications such as antiepileptics, antiretrovirals, antibiotics, and non‐steroidal anti‐inflammatory drugs, can be observed in the setting of ICI therapy due to the reduced immune‐tolerance caused by ICIs. Especially in non‐ICI‐induced SJS/TEN cases, identifying the culprit non‐ICI drug and promptly discontinuing it may permit rechallenge with ICIs in patients with no alternative tumor therapy options.^70^, ^71^, ^72^, ^73^, ^74^ Consequently, the pursuit of the true culprit drug and the exploration of the possibility of excluding ICIs as the cause of SJS/TEN are of critical importance, even in cases that appear to be caused by ICIs. However, it is important to note that even 1 month after discontinuation of ICI therapy, various irAEs, including TEN, can develop due to the prolonged immune enhancement by ICIs.^71^ This underscores the need for special care when considering rechallenge, even in cases where examinations suggest non‐ICI drugs as the cause of irAEs.

In the absence of established diagnostic markers for SJS/TEN, skin biopsy remains a valuable tool for diagnosis and is strongly recommended. When differentiation between SJS/TEN and autoimmune bullous diseases is challenging, immunofluorescence testing can also be helpful. Of note, the overdiagnosis of lichenoid bullous eruptions as SJS/TEN has been reported.^53^ A number of case reports have highlighted the difficulty in distinguishing both severe bullous lichenoid eruptions and autoimmune bullous diseases caused by ICIs from ICI‐induced SJS/TEN, underscoring the importance of conducting histological and immunofluorescence evaluations of lesional skin.^51^, ^53^, ^75^, ^76^, ^77^

The SCORTEN score was unfortunately not consistently documented in the reports analyzed herein. Although the applicability of SCORTEN to ICI‐induced SJS/TEN has yet to be established, more frequent use and reporting of SCORTEN in cases of ICI‐associated SJS/TEN may offer improved evaluation of predicted outcomes. In addition, it would allow for the assessment of the precision of SCORTEN in ICI‐induced SJS/TEN compared to classical SJS/TEN caused by conventional medications. The accumulation of such data might lead to the consideration of developing a specific prognostic score for ICI‐associated SJS/TEN if further data suggest that the SCORTEN score, developed for conventional drugs, proves to be an imprecise tool for predicting the risk of mortality in ICI‐associated SJS/TEN.

In all but one case, ICIs were discontinued to prevent progression and recurrence of the severe cutaneous reaction. This approach sharply contrasts with the management of lichenoid dermatitis caused by ICIs, which, as both common practice and literature reports indicate, is treated with topical steroids without ICI discontinuation in most cases.^25^ In a single case where an ICI was successfully re‐administered, the cause of TEN was attributed to both pembrolizumab and cephalosporin.^29^ Given the high recurrence rate of SJS/TEN on rechallenge with the causative drug, it is possible that TEN in this case was caused by cephalosporin rather than pembrolizumab, as no recurrence occurred following the administration of pembrolizumab.

Systemic corticosteroids are currently the mainstay of therapy for many cutaneous and extracutaneous irAEs,^78^ and the results of this narrative review also demonstrate that systemic corticosteroids are the most common treatment for patients with ICI‐induced SJS/TEN (Figure 3a). Conversely, current management of classical TEN primarily involves supportive care with short‐term pulsed corticosteroids, cyclosporine, high‐dose intravenous immunoglobulin, or TNF antagonists, although controversies remain.^79^, ^80^ While the risks and benefits of systemic corticosteroids for the management of SJS/TEN induced by ICIs should be carefully considered, they can be effective in treating this condition because the underlying mechanism involves a T‐cell mediated immune reaction targeting skin and mucosa triggered by ICIs.

This narrative review highlights the demographics, clinical features and culprit medications of ICI‐associated SJS/TEN. It also identifies gaps in knowledge that should be addressed by further research. These include the identification of molecular markers for prediction of ICI‐associated SJS/TEN occurrence and severity, and the validation or improvement of clinical predictive scoring systems such as SCORTEN for accurate early prediction of outcome in patients suffering from ICI‐associated SJS/TEN. Last but not least, deep molecular phenotyping of biological specimens such as serum, peripheral blood monocytes and skin biopsies of patients affected by ICI‐associated SJS/TEN is required to identify cell types, signaling pathways and druggable molecular targets as a basis for developing improved therapeutic options.

## 5 CONCLUSION

In conclusion, ICI‐related SJS/TEN is a rare but potentially life‐threatening adverse event associated with the use of ICIs. Our narrative review of cases published in the literature provides important insights into the epidemiology, clinical features, and management of this condition. With the increasing use of ICIs, it is crucial to increase understanding about the diagnosis and management of SJS/TEN to improve patient outcomes. Further research is needed to investigate the underlying mechanisms and risk factors associated with ICI‐related SJS/TEN, as well as to develop optimal biomarkers, outcome measurement tools, and management strategies for this condition.

## FUNDING INFORMATION

No funding was received for conducting this study.

## CONFLICT OF INTEREST STATEMENT

Takashi K. Satoh, Matthias Munoz Neulinger, Pia‐Charlotte Stadler, and Rui Aoki have no conflict of interest to declare. Lars E. French has consulted or given lectures for Galderma, Janssen, Leo Pharma, Eli Lilly, Almirall, UNION Therapeutics, Regeneron, Novartis, Amgen, AbbVie, UCB, Biotest, AC Immune, and InflaRx, outside the submitted work.

## ETHICS STATEMENT

This article does not contain any studies involving human participants performed by any of the authors.

## Supporting information


Table S1.

## Data Availability

No additional source data were generated.
