# Supplementary material for: Immune checkpoint inhibitor‐induced epidermal necrolysis: A narrative review evaluating demographics, clinical features, and culprit medications
Source: J Dermatol. 2023 Nov 30;51(1):3–11. doi: 10.1111/1346-8138.17039 (PMC11483933; doi:10.1111/1346-8138.17039)
Supplement: Supplementary file 1 — Table S1. [file JDE-51--s001.docx]

**Supplementary Table 1.**

"Clinical Data of Case Reports with ICI-Induced SJS/TEN"

| Type | Age | Malignancy | ICI | onset (days) | Cycle of onset | BSA (%) | SCO- RTEN | MI | Hist | IF | ST | CoT | Onset to death (days) | Cause of death | Reference |
| --- | --- | --- | --- | --- | --- | --- | --- | --- | --- | --- | --- | --- | --- | --- | --- |
| TEN | NA | lung cancer | Nivo + Ipi | NA | NA | NA | NA | NA | NA | NA | NA | Died | NA | NA | Antonia et al. ^1^ |
| TEN | 64 | melanoma | Nivo | 28 | 2 | NA | NA | No | Yes | Yes | CCS + CsA | Died | 120 | disease progression and sepsis | Nayar et al. ^2^ |
| SJS | 71 | melanoma | Ipi | 60 | 3 | 25 | NA | 3 | Yes | NA | No | Rec. | NA | NA | Pathria et al. ^3^ |
| SJS | 77 | melanoma | Pemb | 3 | 1 | 10 | NA | 1 | Yes | NA | CCS | NA | NA | NA | Goldinger et al. ^4^ |
| TEN | 50 | melanoma | Nivo | NA | NA | 90 | 5 | 3 | Yes | Yes | CCS + IVIg + TNFi | Died | 6 | sepsis | Vivar et al. ^5^ |
| SJS | 75 | lung cancer | Nivo | 25 | NA | NA | NA | NA | NA | NA | CCS | Died | 54 | disease progression | Ichiki et al. ^6^ |
| TEN | 41 | melanoma | Pemb | 14 | 1 | 30 | NA | 2 | NA | NA | No | Died | 10 | NA | Demirtas et al. ^7^ |
| SJS | 76 | lung cancer | Nivo | NA | 2 | NA | NA | 2 | Yes | NA | CCS | Rec | NA | NA | Ito et al. ^8^ |
| SJS | NA | melanoma | Ipi | NA | NA | NA | NA | 1 | Yes | NA | CCS | NA | NA | NA | Dika et al. ^9^ |
| SJS | 50 | HNC | Pemb | 145 | 5 | 4,5 | NA | 2 | Yes | NA | CCS + CsA | Rec. | NA | NA | Saw et al. ^10^ |
| SJS | 53 | renal cell carcinoma | Pemb | 77 | 2 | 3 | NA | 1 | Yes | NA | CsA | Rec. | NA | NA | Saw et al. ^10^ |
| SJS | 63 | HNC | Nivo | 7 | 1 | 8 | NA | 2 | Yes | NA | No | Rec. | NA | NA | Shah et al. ^11^ |
| SJS | 55 | melanoma | Pemb | NA | 9 | NA | NA | 1 | NA | NA | CCS | Rec. | NA | NA | Hwang et al. ^12^ |
| SJS | 59 | lung cancer | Nivo | 21 | 2 | 10 | NA | 2 | NA | NA | CCS | Rec. | NA | NA | Salati et al. ^13^ |
| SJS | 75 | lung cancer | Atez | 57 | 2 | 5 | NA | 2 | Yes | NA | CCS | Rec. | NA | NA | Chirasuthat et al. ^14^ |
| SJS | 69 | lung cancer | Pemb | 17 | 1 | NA | NA | 2 | Yes | NA | CCS | Rec. | NA | NA | Haratake et al. ^15^ |
| TEN | 54 | lymphoma | Nivo | 10 | 1 | 90 | 3 | 1 | NA | NA | CCS | Died | 58 | MOF | Griffin et al. ^16^ |
| TEN | 47 | GI cancer | Nivo + Ipi | 6 | 1 | 54 | 3 | 3 | Yes | NA | CCS | Rec. | NA | NA | Kubicki et al. ^17^ |
| TEN | 63 | lung cancer | Pemb | 4 | 1 | 80 | 3 | 3 | Yes | NA | CCS + CsA | Rec. | NA | NA | Cai et al. ^18^ |
| SJS | 71 | melanoma | Pemb | 43 | 2 | NA | NA | 1 | Yes | NA | CCS | Rec. | NA | NA | Horii et al. ^19^ |
| TEN | 62 | melanoma | Nivo + Ipi | 24 | 2 | 80 | 5 | 1 | Yes | NA | TNFi + CsA | Died | NA | MOF | Logan et al. ^20^ |
| TEN | 78 | melanoma | Pemb | 2 | 1 | NA | 3 | 2 | Yes | NA | CCS | Rec. | NA | NA | Lomax et al. ^21^ |
| SJS | 69 | liver cancer | Nivo | 112 | 8 | NA | NA | 1 | NA | NA | CCS | Rec. | NA | NA | Dasanu et al. ^22^ |
| SJS | 62 | NA | NA | 42 | NA | NA | NA | NA | Yes | NA | CCS + TNFi | Rec. | NA | NA | Coleman et al. ^23^ |
| SJS | 62 | NA | NA | 42 | NA | NA | NA | NA | Yes | NA | CCS + TNFi | Rec. | NA | NA | Coleman et al. ^23^ |
| SJS | 74 | bladder cancer | Pemb | 27 | NA | 10 | NA | 2 | Yes | NA | CCS | Died | NA | disease progression | Hsu et al. ^24^ |
| TEN | 67 | bladder cancer | Atez | 210 | 8 | 80 | NA | 1 | Yes | NA | CCS | Rec. | NA | NA | Hsu et al. ^24^ |
| TEN | 57 | lung cancer | Pemb | 14 | 1 | 80 | NA | 2 | NA | NA | CCS + TNFi | NA | NA | NA | Kumar et al. ^25^ |
| TEN | 62 | melanoma | Nivo + Ipi | 49 | 3 | 30 | 3 | 1 | Yes | Yes | CCS | Rec. | NA | NA | Gopee et al. ^26^ |
| SJS | 80 | lung cancer | Pemb | 105 | 6 | NA | NA | 1 | NA | Yes | CCS | Died | NA | sepsis | Riano et al. ^27^ |
| NOS | 55 | cervical cancer | Pemb | 14 | 1 | NA | NA | 2 | Yes | NA | CCS | Rec. | NA | NA | Robinson et al. ^28^ |
| TEN | 50 | GI cancer | Nivo | 730 | NA | NA | NA | 1 | Yes | NA | CCS + TNFi | Died | NA | acute heart failure | Basu et al. ^29^ |
| NOS | NA | NA | Pemb | NA | NA | NA | NA | NA | NA | NA | NA | NA | NA | NA | Ziemer et al. ^30^ |
| NOS | NA | NA | Pemb | NA | NA | NA | NA | NA | NA | NA | NA | NA | NA | NA | Ziemer et al. ^30^ |
| NOS | NA | NA | Pemb | NA | NA | NA | NA | NA | NA | NA | NA | NA | NA | NA | Ziemer et al. ^30^ |
| NOS | 64 | melanoma | NA | 39 | NA | NA | NA | 2 | Yes | NA | CCS | NA | NA | NA | Molina et al. ^31^ |
| NOS | 86 | NA | NA | 63 | NA | NA | NA | No | Yes | NA | CCS | NA | NA | NA | Molina et al. ^31^ |
| NOS | 80 | lung cancer | NA | 25 | NA | NA | NA | 2 | Yes | NA | CCS | NA | NA | NA | Molina et al. ^31^ |
| NOS | 70 | renal cell carcinoma | NA | 210 | NA | NA | NA | 1 | Yes | NA | CCS | NA | NA | NA | Molina et al. ^31^ |
| NOS | 59 | melanoma | NA | 13 | NA | NA | NA | 2 | Yes | NA | CCS + TNFi | NA | NA | NA | Molina et al. ^31^ |
| NOS | 73 | melanoma | NA | 253 | NA | NA | NA | 1 | Yes | NA | CCS | NA | NA | NA | Molina et al. ^31^ |
| NOS | 34 | lymphoma | NA | 141 | NA | NA | NA | 1 | Yes | NA | CsA | NA | NA | NA | Molina et al. ^31^ |
| SJS | 62 | melanoma | Pemb | 54 | 3 | 10 | NA | No | Yes | Yes | CCS | Rec. | NA | NA | O'Connor et al. ^32^ |
| SJS/TEN overlap | 76 | HNC | Nivo | 27 | 2 | 10 | NA | NA | Yes | NA | CCS + IVIg | Died | 67 | disease progression | Koshizuka et al. ^33^ |
| SJS | 70 | lung cancer | Nivo | 84 | 3 | NA | NA | 1 | Yes | NA | CCS | Rec. | NA | NA | Komatsu et al. ^34^ |
| SJS/TEN overlap | 69 | GI cancer | Pemb | 28 | 1 | 28 | NA | 1 | NA | NA | No | Rec. | NA | NA | Cao et al. ^25^ |
| TEN | 46 | lung cancer | Nivo | NA | NA | NA | NA | NA | NA | NA | NA | NA | NA | NA | Ma et al. ^35^ |
| SJS | 68 | lung cancer | Atez | NA | NA | NA | NA | NA | NA | NA | NA | NA | NA | NA | Ma et al. ^35^ |
| SJS | 64 | lung cancer | Pemb | NA | NA | NA | NA | NA | NA | NA | NA | NA | NA | NA | Ma et al. ^35^ |
| SJS | 51 | melanoma | Nivo + Ipi | NA | NA | NA | NA | NA | NA | NA | NA | NA | NA | NA | Ma et al. ^35^ |
| SJS | 51 | liver cancer | Nivo | NA | NA | NA | NA | NA | NA | NA | NA | NA | NA | NA | Ma et al. ^35^ |
| TEN | 59 | lymphoma | Durv | NA | NA | NA | NA | NA | NA | NA | NA | NA | NA | NA | Ma et al. ^35^ |
| SJS | 46 | lung cancer | Atez | NA | NA | NA | NA | NA | NA | NA | NA | NA | NA | NA | Ma et al. ^35^ |
| SJS | 42 | HNC | Pemb | NA | NA | NA | NA | NA | NA | NA | NA | NA | NA | NA | Ma et al. ^35^ |
| TEN | 86 | liver cancer | Nivo | 28 | 2 | 30 | 3 | 1 | Yes | Yes | CCS + IVIg | Died | 48 | sepsis | Kim et al. ^36^ |
| SJS | 78 | lung cancer | Nivo | NA | 2 | 20 | NA | 2 | Yes | NA | CCS | Rec. | 112 | disease progression | Gracia et al. ^37^ |
| NOS |  | NA |  | NA | NA | NA | NA | NA | NA | NA | NA | NA | NA | NA | He et al. ^38^ |
| SJS | 76 | lung cancer | Pemb | 23 | 1 | 18 | NA | 1 | Yes | NA | CCS + IVIg | Died | 44 | aspiration pneumonia | Oguri et al. ^39^ |
| TEN | 72 | GI cancer | Sint | 7 | 1 | 70 | 5 | NA | Yes | NA | CCS + IVIg | Rec. | NA | NA | Zhao et al. ^40^ |
| TEN | 59 | lung cancer | Sint | 149 | 4 | 95 | NA | 1 | NA | NA | CCS | Rec. | NA | NA | Li et al. ^41^ |
| TEN | 54 | melanoma | Nivo + Ipi | 180 | NA | 90 | 3 | 3 | Yes | NA | CCS + CsA | Rec. | NA | NA | Sommerfelt et al. ^42^ |
| TEN | 34 | renal cell carcinoma | Nivo + Ipi | 5 | 1 | 85 | 3 | 3 | Yes | NA | CCS + CsA | Died | 21 | MOF | Sommerfelt et al. ^42^ |
| SJS | 45 | GI cancer | Nivo | 42 | 3 | 15 | 4 | 1 | Yes | NA | CCS | Rec. | NA | NA | Saad et al. ^43^ |
| NOS | NA | NA |  | NA | NA | NA | NA | NA | NA | NA | NA | NA | NA | NA | Ingen et al. ^44^ |
| TEN | 63 | lung cancer | Pemb | 66 | 3 | 30 | NA | 1 | Yes | NA | CCS + IVIg + CsA | Rec. | 150 | disease progression | Chow et al. ^45^ |
| TEN | 77 | GI cancer | Pemb | 22 | 2 | 90 | 3 | No | Yes | Yes | CCS + CsA | Rec. | NA | NA | Gallo et al. ^46^ |
| SJS | 64 | bladder cancer | Pemb | 4 | 1 | NA | NA | 1 | NA | NA | CCS | Rec. | NA | NA | Ryu et al. ^47^ |
| SJS | 68 | lung cancer | Pemb | 26 | 1 | 45 | 4 | 2 | NA | NA | CCS + TNFi | Rec. | NA | NA | Wu et al. ^48^ |
| SJS | 32 | liver cancer | Pemb | 110 | 3 | 8 | NA | NA | Yes | NA | CCS + TNFi | Rec. | 7 | disease progression | Zhang et al. ^49^ |
| TEN | 65 | lung cancer | Pemb | 4 | 1 | NA | NA | NA | Yes | NA | CCS + IVIg | Rec. | NA | NA | Kian et al. ^50^ |
| TEN | 60 | lung cancer | Pemb | 26 | 2 | 80 | 3 | 1 | Yes | Yes | CCS + IVIg | Rec. | NA | NA | Alexandris et al. ^51^ |
| TEN | 70 | lung cancer | Atez | 30 | 2 | 75 | 6 | 1 | Yes | Yes | CCS | Died | 10 | ARDS | Alexandris et al. ^51^ |
| SJS | NA | NA | Pemb | 28 | NA | NA | NA | NA | NA | NA | NA | NA | NA | NA | Cho et al. ^52^ |
| SJS | NA | NA | Pemb | 42 | NA | NA | NA | NA | NA | NA | NA | NA | NA | NA | Cho et al. ^52^ |
| TEN | 67 | HNC | Pemb | 31 | 2 | NA | NA | 2 | Yes | NA | CCS | Died | 6 | NA | Golle et al. ^53^ |
| SJS | 68 | lung cancer | Pemb | 21 | 1 | NA | NA | 3 | NA | NA | CCS | Rec. | NA | NA | Machida et al. ^54^ |
| NOS | 27 | HNC | Sint | 56 | 3 | NA | NA | 2 | Yes | NA | CCS + IVIg | Rec. | NA | NA | Huang et al. ^55^ |

**Abbreviations:**

NOS: Not otherwise specified

HNC: head and neck cancer

GI: gastrointestinal

Nivo: Nivolumab

Ipi: Ipilimumab

Pemb: Pembrolizumab

Atez: Atezolizumab

Durv: Durvalumab

Sint: Sintilimab

MI: Number of mucosal involvenment

Hist: Histological evaluation

IF: Immunoﬂuorescence

ST: Systemic treatment

CCS: Corticosteroid

TNFi: TNF inhibitor

CsA: Cyclosporine A

CoT: Consequences of treatment

Rec.: Recovered

MOF: Multiple organ failure

**References**

1. Antonia, S. J. *et al.* Safety and Efficacy of First-Line Nivolumab (Anti-PD-1; BMS-936558, ONO-4538) and Ipilimumab in Non-Small Cell Lung Cancer (NSCLC) Metastatic Non-Small Cell Lung Cancer. *Int J Radiat Oncol Biology Phys* **90**, S32–S33 (2014).

2. Nayar, N., Briscoe, K. & Penas, P. Toxic Epidermal Necrolysis–like Reaction With Severe Satellite Cell Necrosis Associated With Nivolumab in a Patient With Ipilimumab Refractory Metastatic Melanoma. *J Immunother* **39**, 149–152 (2016).

3. Pathria, M., Mundi, J. & Trufant, J. A case of Stevens–Johnson syndrome in a patient on ipilimumab. *Int J Case Reports Images* **7**, 300 (2016).

4. Goldinger, S. M. *et al.* Cytotoxic Cutaneous Adverse Drug Reactions during Anti-PD-1 Therapy. *Clinical Cancer Research* **22**, 4023–4029 (2016).

5. Vivar, K. L. *et al.* Epidermal programmed cell death-ligand 1 expression in TEN associated with nivolumab therapy. *J Cutan Pathol* **44**, 381–384 (2017).

6. Ichiki, Y. *et al.* [Analysis of Advanced or Postoperative Recurrent Non-small Lung Cancer Cases Treated with Nivolumab]. *J UOEH* **39**, 291–297 (2017).

7. Demirtas, S., El Aridi, L., Acquitter, M., Fleuret, C. & Plantin, P. [Toxic epidermal necrolysis due to anti-PD1 treatment with fatal outcome]. *Ann Dermatol Venereol* **144**, 65–66 (2017).

8. Ito, J. *et al.* Aprepitant for refractory nivolumab-induced pruritus. *Lung Cancer* **109**, 58–61 (2017).

9. Dika, E. *et al.* Cutaneous adverse effects during ipilimumab treatment for metastatic melanoma: a prospective study. *Eur J Dermatol* **27**, 266–270 (2017).

10. Saw, S., Lee, H. Y. & Ng, Q. S. Pembrolizumab-induced Stevens-Johnson syndrome in non-melanoma patients. *Eur J Cancer* **81**, 237–239 (2017).

11. Shah, K. M., Rancour, E. A., Al-Omari, A. & Rahnama-Moghadam, S. Striking enhancement at the site of radiation for nivolumab-induced Stevens-Johnson syndrome. *Dermatology Online J* **24**, (2018).

12. Hwang, A., Iskandar, A. & Dasanu, C. A. Stevens-Johnson syndrome manifesting late in the course of pembrolizumab therapy. *J Oncol Pharm Pract* **25**, 1520–1522 (2018).

13. Salati, M. *et al.* Stevens-Johnson syndrome during nivolumab treatment of NSCLC. *Ann Oncol* **29**, 283–284 (2018).

14. Chirasuthat, P. & Chayavichitsilp, P. Atezolizumab-Induced Stevens-Johnson Syndrome in a Patient with Non-Small Cell Lung Carcinoma. *Case Reports Dermatology* **10**, 198–202 (2018).

15. Haratake, N. *et al.* Stevens-Johnson Syndrome Induced by Pembrolizumab in a Lung Cancer Patient. *J Thorac Oncol* **13**, 1798–1799 (2018).

16. Griffin, L. L. *et al.* Toxic epidermal necrolysis (TEN) associated with the use of nivolumab (PD-1 inhibitor) for lymphoma. *Jaad Case Reports* **4**, 229–231 (2018).

17. Kubicki, S. L., Welborn, M. E. & Patel, A. B. Toxic Epidermal Necrolysis During Cotherapy with Ipilimumab and Nivolumab. *J Immunother Precis Oncol* **1**, 78–81 (2018).

18. Cai, Z. R. *et al.* Toxic epidermal necrolysis associated with pembrolizumab. *J Oncol Pharm Pract* **26**, 1259–1265 (2019).

19. Horii, M., Kobayashi, T., Maeda, S., Takehara, K. & Matsushita, T. Stevens-Johnson syndrome associated with radiation recall dermatitis in a patient treated with immune checkpoint inhibitor. *J Dermatol* **46**, e434–e436 (2019).

20. Logan, I. T., Zaman, S., Hussein, L. & Perrett, C. M. Combination Therapy of Ipilimumab and Nivolumab-associated Toxic Epidermal Necrolysis (TEN) in a Patient With Metastatic Melanoma: A Case Report and Literature Review. *J Immunother Hagerstown Md 1997* 1 (2019) doi:10.1097/cji.0000000000000302.

21. Lomax, A. J., McQuillan, P. I. A., Hall, A. & McArthur, G. A. Acute toxic epidermal necrolysis reaction post single dose pembrolizumab with preceding cephalosporin exposure: successful rechallenge with anti-PD-1 therapy. *Intern Med J* **49**, 1051–1053 (2019).

22. Dasanu, C. A. Late-onset Stevens-Johnson syndrome due to nivolumab use for hepatocellular carcinoma. *J Oncol Pharm Pract* **25**, 2052–2055 (2019).

23. Coleman, E. *et al.* Inflammatory eruptions associated with immune checkpoint inhibitor therapy: A single-institution retrospective analysis with stratification of reactions by toxicity and implications for management. *J Am Acad Dermatol* **80**, 990–997 (2019).

24. Hsu, T.-J. & Liu, K.-L. Stevens–Johnson syndrome and toxic epidermal necrolysis related to immune checkpoint inhibitors: Two cases and literature review. *Dermatol Sin* **38**, 236 (2020).

25. Cao, J. *et al.* Pembrolizumab-induced autoimmune Stevens-Johnson syndrome/toxic epidermal necrolysis with myositis and myocarditis in a patient with esophagogastric junction carcinoma: a case report. *Transl Cancer Res* **0**, 0–0 (2021).

26. Gopee, N. H., Gourley, A. M., Oliphant, T. J. & Hampton, P. J. Toxic epidermal necrolysis occurring with immune checkpoint inhibitors. *Dermatology Online J* **26**, (2020).

27. Riano, I., Cristancho, C. & Treadwell, T. Stevens-Johnson Syndrome-Like Reaction After Exposure to Pembrolizumab and Recombinant Zoster Vaccine in a Patient With Metastatic Lung Cancer. *J Investig Med High Impact Case Rep* **8**, 2324709620914796 (2020).

28. Robinson, S., Saleh, J., Curry, J. & Mudaliar, K. Pembrolizumab-Induced Stevens-Johnson Syndrome/Toxic Epidermal Necrolysis in a Patient With Metastatic Cervical Squamous Cell Carcinoma: A Case Report. *Am J Dermatopathol* **42**, 292–296 (2020).

29. Basu, P., Tong, Y., Hinds, B. R. & Schneider, J. A. Nivolumab-induced toxic epidermal necrolysis with retiform purpura. *Br J Dermatol* **183**, e32 (2020).

30. Ziemer, C. M. *et al.* Immunohistochemical Expression of PD-L1 Is Increased in Lesional Epidermal Keratinocytes in Stevens-Johnson Syndrome/Toxic Epidermal Necrolysis. *Am J Dermatopathol* **43**, 318–320 (2021).

31. Molina, G. E., Yu, Z., Foreman, R. K., Reynolds, K. L. & Chen, S. T. Generalized bullous mucocutaneous eruption mimicking Stevens-Johnson syndrome in the setting of immune checkpoint inhibition: A multicenter case series. *J Am Acad Dermatol* **83**, 1475–1477 (2020).

32. O’Connor, C., Jordan, E. & O’Connell, M. Atypical Stevens–Johnson syndrome caused by pembrolizumab in the treatment of metastatic melanoma – Are corticosteroids a safe treatment option? *J Dermatology Dermatologic Surg* **24**, 137 (2020).

33. Koshizuka, K. *et al.* Toxic epidermal necrolysis associated with nivolumab treatment for head and neck cancer. *Clin Case Reports* **9**, 848–852 (2021).

34. Komatsu-Fujii, T., Ogawa, M., Nonoyama, S., Fukumoto, T. & Tanabe, H. Recurrence of nivolumab-induced Stevens-Johnson syndrome due to tegafur/gimeracil/oteracil (TS-1®) after nivolumab discontinuation. *Eur J Dermatol* **31**, 98–99 (2021).

35. Ma, K. S.-K. *et al.* Ocular manifestations of anti-neoplastic immune checkpoint inhibitor-associated Stevens-Johnson syndrome/toxic epidermal necrolysis in cancer patients. *Ocular Surf* **22**, 47–50 (2021).

36. Kim, M. C. & Khan, H. N. Nivolumab-Induced Toxic Epidermal Necrolysis: Rare but Fatal Complication of Immune Checkpoint Inhibitor Therapy. *Cureus* **13**, e15017 (2021).

37. Gracia-Cazaña, T., Padgett, E., Calderero, V. & Oncins, R. Nivolumab-associated Stevens-Johnson syndrome in a patient with lung cancer. *Dermatology Online J* **27**, (2021).

38. He, C., Si, X., Zhang, L., Xu, Y. & Qu, T. Immune checkpoint inhibitor-related epidermal necrolysis: A rare condition with poor prognosis. *Eur J Cancer* **145**, 194–196 (2021).

39. Oguri, T. *et al.* A Case of Guillain-Barré Syndrome and Stevens-Johnson Syndrome/Toxic Epidermal Necrosis Overlap After Pembrolizumab Treatment. *J Investigative Medicine High Impact Case Reports* **9**, 23247096211037464 (2021).

40. Zhao, Y., Cao, Y., Wang, X. & Qian, T. Treatment of PD-1 Inhibitor-Associated Toxic Epidermal Necrolysis: A Case Report and Brief Review. *Oncotargets Ther* **15**, 345–351 (2022).

41. Li, G., Gong, S., Wang, N. & Yao, X. Toxic epidermal necrolysis induced by sintilimab in a patient with advanced non-small cell lung cancer and comorbid pulmonary tuberculosis: A case report. *Front Immunol* **13**, 989966 (2022).

42. Sommerfelt, H. *et al.* Toxic epidermal necrolysis after immune checkpoint inhibition, case report, and review of the literature. *Acta Oncol* **61**, 1295–1299 (2022).

43. Saad, E. *et al.* Steven-Johnson Syndrome: A Rare but Serious Adverse Event of Nivolumab Use in a Patient With Metastatic Gastric Adenocarcinoma. *J Medical Cases* **13**, 449–455 (2022).

44. Ingen-Housz-Oro, S. *et al.* Severe blistering eruptions induced by immune checkpoint inhibitors: a multicentre international study of 32 cases. *Melanoma Res* **32**, 205–210 (2022).

45. Chow, K. V. C., O’Leary, C., Paxton-Hall, F., Lambie, D. & O’Byrne, K. Pembrolizumab-induced toxic epidermal necrolysis: case report. *Oxf Medical Case Reports* **2022**, omac025 (2022).

46. Gallo Marin, B. *et al.* Pembrolizumab-induced Toxic Epidermal Necrolysis in a Patient with Metastatic Esophageal Adenocarcinoma. *R I Med J (2013)* **105**, 34–36 (2022).

47. Ryu, S., Jun, I., Kim, T.-I., Seo, K. Y. & Kim, E. K. Pembrolizumab-induced Stevens-Johnson Syndrome with Severe Ocular Complications. *Ocul Immunol Inflamm* **30**, 1533–1535 (2022).

48. Wu, J.-Y., Kang, K., Yi, J. & Yang, B. Pembrolizumab-induced Stevens-Johnson syndrome in advanced squamous cell carcinoma of the lung: A case report and review of literature. *World J Clin Cases* **10**, 6110–6118 (2022).

49. Zhang, J. *et al.* Pembrolizumab associated Stevens-Johnson syndrome with porokeratosis in a patient in the setting of primary hepatocellular carcinoma. *Australas J Dermatol* **63**, e71–e74 (2022).

50. Kian, W. *et al.* Intravenous immunoglobulin efficacy on pembrolizumab induced severe toxic epidermal necrolysis. *Anti-cancer Drug* **33**, e738–e740 (2022).

51. Alexandris, D., Alevizopoulos, N., Gakiopoulou, H., Stavrinou, N. & Vourlakou, C. Cutaneous Stevens Johnson - Toxic Epidermal Necrolysis Immunotherapy related Toxicities in Lung Cancer Patients. *J Oncol Pharm Pract* **28**, 1276–1282 (2022).

52. Cho, Y.-T., Lin, Y.-T., Yang, C.-W. & Chu, C.-Y. Cutaneous immune-related adverse events among Taiwanese cancer patients receiving immune checkpoint inhibitors link to a survival benefit. *Sci Rep-uk* **12**, 7021 (2022).

53. Golle, L., Michl, C. & Kreft, B. [Bullous drug reaction after pembrolizumab administration: two case reports]. *Dermatologie (Heidelb)* **73**, 959–964 (2022).

54. Machida, M. *et al.* A case report involving suppressed nuclear receptor transcription factors 4a1 and Stevens-Johnson syndrome induced by a single dose of pembrolizumab and successfully treated with early steroid administration, resulting in complete remission of stage III lung cancer. *J Pharm Heal Care Sci* **8**, 29 (2022).

55. Huang, Y. *et al.* A case of sintilimab-induced SJS/TEN:Dermatologic adverse reactions associated with programmed cell death protein-1 inhibitors. *Dermatol Ther* **35**, e15663 (2022).
